# Protein Drugs Related to Allergic Reaction

**DOI:** 10.1155/2015/614048

**Published:** 2015-05-11

**Authors:** Ji-Fu Wei, Tian-Rui Xu, Ming-Can Yu, Xing-Ding Zhou, Zuo-Tao Zhao, Yang Jin

**Affiliations:** ^1^Research Division of Clinical Pharmacology, the First Affiliated Hospital of Nanjing Medical University, Nanjing, Jiangsu 210029, China; ^2^Cell Signalling Laboratory, Faculty of Life Science and Technology, Kunming University of Science and Technology, Kunming, Yunnan 650500, China; ^3^Division of Endocrinology, Metabolism and Lipids, Emory University School of Medicine, Atlanta, GA 30322-0001, USA; ^4^Ngee Ann Polytechnics, Singapore 599489; ^5^First Hospital, Peking University, Beijing 100034, China; ^6^Department of Biosciences, University of Oslo, 0407 Oslo, Norway

Allergic reactions are sensitive to substances called allergens that come into contact with the skin, nose, eyes, respiratory tract, and gastrointestinal tract. Many allergic reactions are mild, while others can be severe and life threatening. Protein drugs related to allergic reaction include two categories. The first is protein drugs that are used to treat or prevent allergic reaction. Compared to other small molecule drugs, pharmaceutical peptides and proteins offer low toxicity and high specificity and demonstrate fewer toxicology issues. The second is protein drugs which are used to treat other disease and have the adverse effect causing serious allergic reaction. In this respect, this special issue will add a few new points in the picture of protein drugs related to allergic reaction.

C. Xu et al. in “Efficacy of Sublingual Immunotherapy with* Dermatophagoides farinae* Extract in Monosensitized and Polysensitized Patients with Allergic Rhinitis: Clinical Observation and Analysis” investigated differences in the efficacy of sublingual immunotherapy (SLIT) with* Dermatophagoides farinae* drops in monosensitized and polysensitized allergic rhinitis patients. They divided the patients into two groups: monosensitized group (*n* = 20) and a polysensitized group (*n* = 30). Total nasal symptom scores of patients before and after SLIT were analyzed to evaluate the curative effect. The phylogenetic tree of dust mite allergens as well as other allergens that were tested by skin prick was constructed to help the analysis. They concluded that there was no significant difference in the efficacy of SLIT between dust mite monosensitized and polysensitized patients. Both dust mite monosensitized and polysensitized patients could be cured by SLIT using* Dermatophagoides farinae* drops. This study provides a reference for the selection of allergens to use in immunotherapy for polysensitized AR patients.

In 1997, the first monoclonal antibody (MoAb), the chimeric anti -CD20 molecule rituximab, was approved by the US Food and Drug Administration for use in cancer patients. Since then, the panel of MoAbs that are approved by international regulatory agencies for the treatment of hematopoietic and solid malignancies has continued to expand, currently encompassing a stunning amount of 20 distinct molecules for 11 targets. M. Guan et al. in “Adverse Events of Monoclonal Antibodies Used for Cancer Therapy” provided a brief scientific background on the use of MoAbs in cancer therapy, reviewed all types of monoclonal antibodies-related adverse events (e.g., allergy, immune-related adverse events, cardiovascular adverse events, and pulmonary adverse events), and discussed the mechanism and treatment of adverse events.

To analyze the clinical characteristics of inpatients anaphylaxis and the factors that influenced those characteristics, R. Tang et al. in “Clinical Characteristic of Inpatients with Anaphylaxis in China” collected the patient records from 1990 to 2013 from three highly ranked Chinese hospitals and retrospectively analyzed the characteristics of 108 inpatients anaphylaxis. The mean patient age was 42 ± 20 years old with a male-to-female ratio of 1 : 1.3. The most common trigger was medications (97/108). The most common clinical manifestations included cutaneous (72.2%), nervous (54.6%), respiratory (52.8%), circulatory (41.7%), and digestive (38.0%) signs and symptoms. Male patients were more likely to experience loss of consciousness. Epinephrine was used as the first-line treatment for 56 cases. Inpatient anaphylaxis was more common in female patients and the number increased gradually during the study period.

Y.-J. Guo et al. in “Analysis of Anaphylactic Shock Caused by 17 Types of Traditional Chinese Medicine Injections Used to Treat Cardiovascular and Cerebrovascular Diseases” described anaphylactic shock following treatment of cardiovascular and cerebrovascular diseases with Chinese herbal injections were described. Their analysis of these reports showed that anaphylactic shock caused by Traditional Chinese Medicine (TCM) injections for the treatment of cardiovascular and cerebrovascular diseases is common but also sometimes fatal. They then proposed the following four suggestions for improving the clinical safety of delivering Chinese herbal injections and reducing the occurrence of allergic shock.

J.-M. Yu in “Diversity of House Dust Mite Species in Xishuangbanna Dai, a Tropical Rainforest Region in Southwest China” enrolled the mite-allergic patients, who visited the Xishuangbanna Dai Autonomous Prefecture Hospital from August 2010 to January 2011 and collected the dust samples from the patients' homes by vacuuming. They isolated the mites in this samples by the flotation method and underdone the morphologically based species determination. In total, 6316 mite specimens of morphologically identifiable species were found in 233 dust samples taken from 41 homes. It showed that the mite family of Pyroglyphidae occupied the highest percentage (96.6%) of the total amount of mites collected, followed by Cheyletidae family (2.0%). The most common adult Pyroglyphidae mites were* Dermatophagoides farinae *(76.5%). The most common mites found besides Pyroglyphidae were* Blomia tropicalis*,* Tyrophagus putrescentiae*, and* Aleuroglyphus ovatus*, which were found in 24.4%, 24.4%, and 7.3% of homes, respectively.

*Ji-Fu Wei* *Tian-Rui Xu* *Ming-Can Yu* *Xing-Ding Zhou* *Zuo-Tao Zhao* *Yang Jin*

